# Melange

**Published:** 1885-08

**Authors:** 


### Melange.

That Male Uterus turns out (as we supposed) to have been a typographical error. We must caution our esteemed contemporary to be more careful with his proof. Such accidents are, though, liable to happen to the most careful. He has our sympathy, and although the following explanation spoils our article in last month's issue, we reproduce it with pleasure, and as a matter of simple justice to our neighbor:

"Correction.—In the July issue of The Journal, page 277, an absurd typographical error occurs. The error occurs in line 28, where No. 2 should read *No.* 1. Line 29, male should read *mole*. The phrase corrected should read: 'I detached and removed it, and uterus No. 1 contracted, forcing out my hand with what I took to be a mole. Uterus No. 2 was then well contracted, and both could be felt through the walls of the abdomen, distinctly separated and lying side by side.' "—*Atlanta Med. and Surg. Journal.*

Give Us Your Names Gentlemen.—It is to be observed that the members of the Dallas County (Tex.) Medical Association who passed the resolutions published in our department of Society Notes, did not sign their names, as have all the dissenters who have preceeded them. This, we regard, as unfortunate. We question the right of even a majority of the members of a society to take such action in the name of the society without affixing the names of all who participated in the movement. Otherwise, a bare quorum could bind the entire association in an act which, perhaps, a majority of members if present would vote down. It is something like the cross-road politician's pronunciamento beginning: "*We*, the people of Texas." Give us your names gentlemen, for publication, that your Philadelphia friends may duly applaud your pluck, and acknowledge your "sympathy."

---

False Hopes.—Let not the Communist Medical Journals lay the flattering unction to their souls that the American Medical Association will rescind its action taken at New Orleans, and thus put in a plea of idiocy; were it to do so, it would well deserve some of the bitter denunciation and abuse heaped upon it by those Journals. And there will be a Medical Congress, the howlings organs notwithstanding. It has been said that what America wants is a "National Association of Scientific Medicine," meaning a concern without a code of ethics, we suppose. Very good; let the dissatisfied ones who so pride themselves on their "science," organize such association. It would be like a highly educated woman without virtue, or a beautiful flower without fragrance. The Code of Ethics of American Medicine is its *sine qua non* to respectability, as it is to its integrity and coherence.

---

Acknowledgment.—The overwhelming favor with which the first number of this Journal has been received everywhere, evidences of which have poured in, in the way of congratulations, wishes for, and prophesies of success, and accompanied in the majority of cases by cash subscriptions, argues well for the future. The demand for "sample copies" has been unprecedented, and our large July edition is exhausted.

We wish to impress upon the profession the importance of subscribing *now* in order to get complete files ; it will be impossible *to furnish back numbers*. We know the scarcity of money is very general, but surely $2.00 can be raised ; and once paid, it will not be felt. Send it now—*we need it*—if you would have us succeed, and receive every month this bright, cheerful and instructive little visitor which, like a child, will steal into your affections if you will give it encouragement.

OUR NEXT NUMBER will be enriched by several valuable contributions by men whose names command respect wherever mentioned. One by Dr. T. C. Osborn, of Cleburne. Dr. Osborn was unanimously elected an honorary member of the Texas Medical Association, at its last meeting, on account of his age and high standing in the profession, his professional services, and his contributions to medical literature, which have been numerous and valuable.

One by Dr. Thos. D. Wooten, of Austin, which was read before the Travis County Medical and Surgical Association and requested for publication, by resolution of the Society, in this Journal. And one, also, by Dr. J. W. McLaughlin, of Austin, on Tubercle. Dr. McLaughlin's report of a case of Septic Salpingitis, published in the *Texas Courier Record*, has been extensively copied by other Journals. We are much gratified to see it reproduced entire in Gaillard's Journal for July.

